# Preserving self-renewal of porcine pluripotent stem cells in serum-free 3i culture condition and independent of LIF and b-FGF cytokines

**DOI:** 10.1038/s41420-017-0015-4

**Published:** 2018-02-14

**Authors:** Yangyang Ma, Tong Yu, Yuanxing Cai, Huayan Wang

**Affiliations:** Department of Animal Biotechnology, College of Veterinary Medicine, Northwest A&F University, Yangling, Shaanxi China

## Abstract

Derivation of bona fide porcine pluripotent stem cells is still a critical issue because porcine embryonic stem cells (ESCs) are not available yet, and most of the culture conditions to maintain porcine induced pluripotent stem cells (piPSCs) are based on conditions for mouse and human iPS cells. In this study, we generated a doxycycline-inducible porcine iPS cell line (DOX-iPSCs) and used it to screen the optimal culture condition to sustain the self-renewal of piPSCs. We found that LIF and b-FGF were required for porcine cell reprogramming, but were not essential cytokines for maintaining the self-renewal and pluripotency of piPSCs. A serum-free 3i medium, which includes three inhibitors CHIR99021, SB431542, and PD0325901, three cytokines BMP4, SCF, and IL-6, and human platelet lysates (PL), was made through serious selections. In 3i condition, the doxycycline-inducible iPSCs could be passaged for a long term without the addition of doxycycline, and the flattened morphology of intermediate state piPSCs could convert to the naïve-like morphology with the increase in endogenous pluripotent gene expressions. Additionally, pPSC cell line isolated from 5.5 days blastocysts could be sustained in 3i medium and the expression of endogenous pluripotent genes *OCT4*, *ESRRB*, and *STELLA* was significantly increased. Our finding directed a new reprogramming strategy by using 3i condition to maintain and convert primed piPSCs into naïve-like pluripotent state. A combination of traditional LIF/b-FGF conditions and 3i condition may help us to find out an appropriate reprogramming approach to generate the naïve state of porcine iPSCs.

## Introduction

Somatic cells can be reprogramed by the ectopic expression of defined transcription factors^1,2^. Genetic individuality indicates that the resultant induced pluripotent stem cells (iPSCs) reserved from precursor cells enable personalized cell therapy and regenerative medicine^3^. Pig is an ideal animal model for regenerative medicine due to its close resemblance to humans in body size, physical structure, and metabolism^4,5^. The derivation of porcine iPSCs could not only broaden the platform of pre-clinical trials for human diseases^6^, but also provided a potential carrier for human organ production with less ethical questions^7^.

With substantial improvement in the reprogramming approach, iPSCs were proved indistinguishable from embryonic stem cells (ESCs)^8–11^. Therefore, the fundamental issue in animal species, including pig, is how to fully convert the somatic cells into ESC-like and germline-competent pluripotent stem cells (PSCs). Many efforts have been made to obtain the authentic porcine PSCs referring to the pluripotent criteria that were based on mouse ESCs or iPSCs, including gain of multiple differentiation capacities in vivo and in vitro^12,13^, long term single-cell passages^13–15^, double activated X chromosomes^16,17^, derivation of chimeric fetus^17^, and even chimeric offspring^14^. However, the pluripotent states of the reported porcine iPSC (piPSC) lines were varied because they were derived from different culture conditions with leukemia inhibitory factor (LIF)-dependent^18,19^, basic fibroblast growth factor (b-FGF)-dependent^6,14^, or even both LIF- and b-FGF-dependent media^20^. Thus, the question is whether there is a unique culture condition and regulatory circuitry, which is specific for maintaining piPSCs, and may be different from the signaling pathways used for maintaining human and mouse PSCs^21,22^.

The fully reprogrammed pluripotency can be sorted into ICM-like state (naïve) and post-implantation epiblasts state (primed)^23^. Dissections of each pluripotent state indicated that the naïve state was dependent on JAK/STAT signaling that was activated by LIF, and the primed state was dependent on PI3K/AKT and ALK/SMADs signaling that was activated by b-FGF and transforming growth factor-β1 (TGF-β1)/Activin A. The primed state pluripotency in human and mouse PSCs showed similar gene expression profiles and culture requirements^24–26^; however, the naïve pluripotency was different between the two species, which required different stimulations^24,27–30^. Unfortunately, both defined states were illusive in pig since none of the above conditions were capable of deriving fully reprogrammed porcine ESCs^31^. The species-related regulatory signaling pathway as reported in mouse and human PSCs is likely to be applied in pig and other animals^32^, in which PI3K/AKT and TGF-beta signaling pathways, instead of LIF and b-FGF signaling pathways, may play key roles in maintaining porcine stem cell pluripotency^33,34^. Consequently, a composition of different stimulations may be required for the derivation of porcine PSCs that meet all the criteria of authentic pluripotency.

Studies showed that LIF was dispensable for the derivation of pluripotency^32^. Self-renewal and pluripotency of mouse PSCs were enabled by the elimination of differentiation-inducing signaling of mitogen-activated protein kinase (MAPK) and additional inhibition of glycogen synthase kinase 3 (GSK3), consolidated biosynthetic capacity, and suppressed residual differentiation^32^. For converting the primed human PSCs to the naïve state, additional pathways were required to be blocked besides the above described cultural conditions^27–29^. Accordingly, the proper elimination of differentiation-inducing signaling pathways during porcine cell reprogramming may elevate the pluripotent state and promote the efficacy in generating porcine PSCs.

To optimize the piPSC culture conditions, we established a doxycycline-inducible porcine iPS cell line (DOX-iPSCs) and used it to screen the optimal culture condition to sustain the self-renewal of piPSCs. By screening different extrinsic cytokines that promote different signaling pathways and small molecules that suppress differentiation signals, a 3i culture medium that was serum free and independent on LIF and b-FGF pathways was made and used to maintain the piPSCs.

## Results

### Characterization of doxycycline-inducible porcine iPS cells

The previous reports of piPSCs showed that the incomplete silence of transgenes caused the reprogrammed iPSCs to stay in an inadequate pluripotent state^6,17,35^. Thus, to set a doxycycline-inducible piPSCs, in which expression of the transgenes can be completely switched on/off by doxycycline (Dox), lentiviral particles of TetO-FUW-OSKM and FUW-M2rtTA were infected into porcine embryonic fibroblasts (PEFs) to reprogram the somatic cells into doxycycline-inducible porcine iPS cells (DOX-iPSCs) (Supplementary Fig. 1A). The differences between DOX-iPSCs and previous reported doxycycline-inducible porcine iPF4-2 were the usages of different cultural media, transcription factors, and vectors^13,21^. Three DOX-iPS cell lines (A1, B2, and D1) were generated and cultured in a defined LF2i medium supplemented with LIF, b-FGF, CHIR99021, SB431542, and 4 μg/mL Dox. The DOX-iPS cell colonies showed the domed morphology with sharp-edged border, the positive staining of alkaline phosphatase (AP), and the high-level expression of pluripotent genes (Supplementary Fig. 1A-B). The results of immunofluorescence staining demonstrated that the pluripotent markers OCT4, SOX2, and SSEA-1 were highly expressed in all three piPS cell lines, but NANOG expression was low (Supplementary Fig. 1C). These DOX-iPS cell lines had normal karyotype with 38 chromosomes (Supplementary Fig. 1D), and could form embryoid bodies in vitro and spontaneously differentiate into three germ layers (Supplementary Fig. 1E-F). Since the three DOX-iPS cell lines retain the similar self-renewal and pluripotent features, the cell line A1 of DOX-iPSCs was selected for the following studies.

**Fig. 1:**
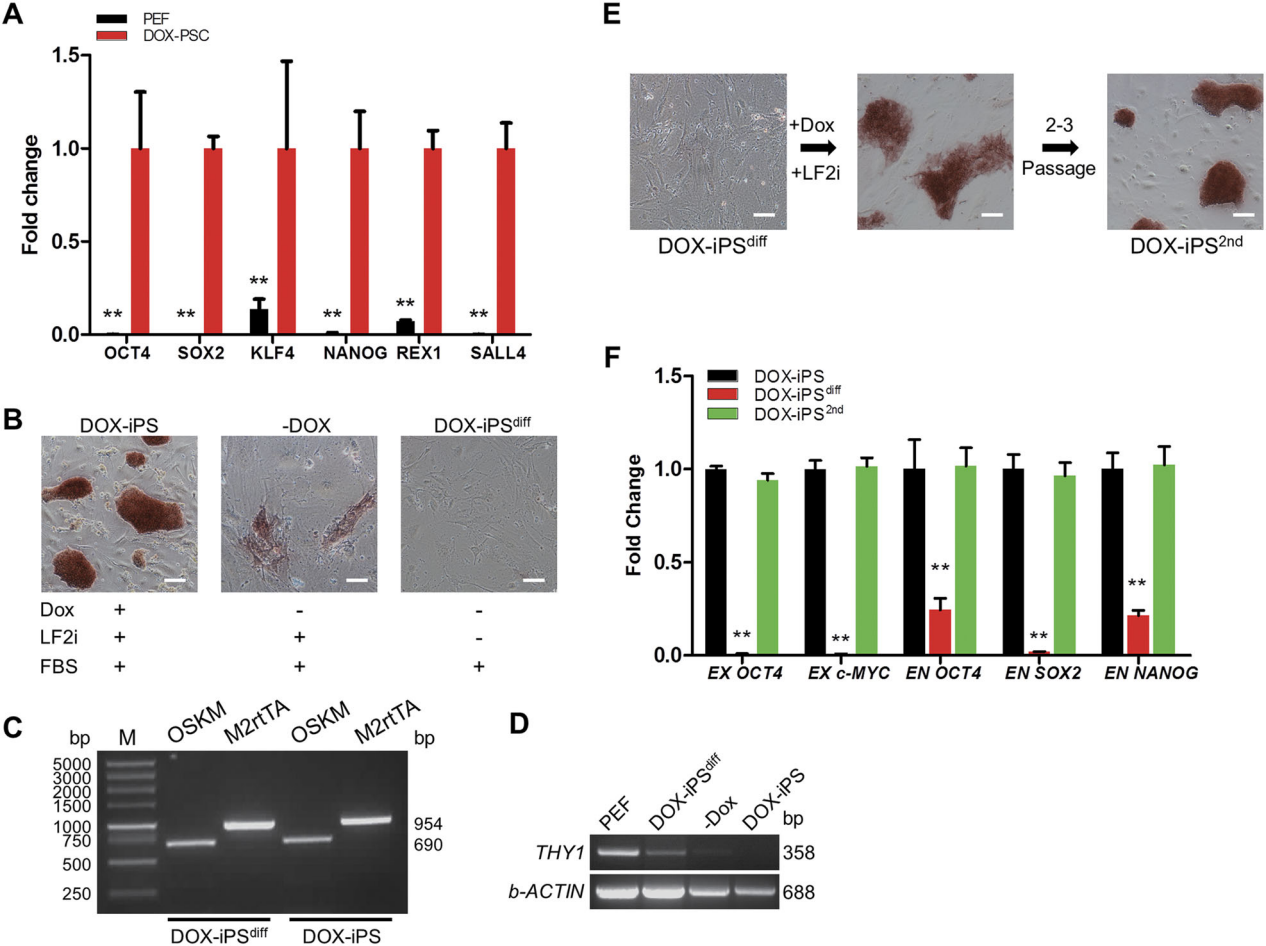
Characterization of doxycycline-inducible porcine iPS cells. The DOX-iPSCs were cultured in LF2i medium with or without doxycycline. **a** Quantitative RT-PCR analysis of pluripotent genes in DOX-iPSCs and PEF cells. **b** Alkaline phosphatase (AP) staining of DOX-iPSCs and the differentiated DOX-iPS (DOX-iPS^diff^) cells. **c** PCR analysis of transgenes from TetO-FUW-OSKM and FUW-M2rtTA in DOX-iPSCs and DOX-iPS^diff^ cells. **d** RT-PCR analysis of *THY 1* expression in PEF, DOX-iPS^diff^ cells, and DOX-iPSCs. –Dox, cells were cultured without the addition of Dox. *b-ACTIN* was as internal control. **e** DOX-iPS^diff^ cells were reprogrammed in the medium with LF2i and Dox for 2–3 passages. The cells derived from DOX-iPS^diff^ cells were named DOX-iPS ^2nd^ cells and were stained for AP. **f** Quantitative RT-PCR analysis of transgenes (EX) and endogenous pluripotent genes (EN) in DOX-iPSCs, DOX-iPS^diff^, and DOX-iPS^2nd^ cells. Scale bar, 100 μm. Data indicate mean ± SD; ** *P* < 0.01, *n* = 3

In DOX-iPSCs, the expression level of transgenes was significantly increased versus the control PEF cells. The expression of endogenous pluripotent genes *NANOG*, *REX1*, and *SALL4* was also significantly activated in DOX-iPSCs (Fig. 1a). However, as soon as Dox was withdrawn, the AP staining of DOX-iPSCs was faded, and the morphology was flattened, indicating that the culture condition without Dox was insufficient to maintain the pluripotent state of DOX-iPSCs (Fig. 1b). Upon culturing DOX-iPSCs in a Dox-free medium without all cytokines and small molecules for 4–5 days, the cells attained a fibroblast-like morphology and were negative for AP (Fig. 1b). The differentiated DOX-iPS (DOX-iPS^diff^) cells still retained the insertions of TetO-FUW-OSKM and FUW-M2rtTA (Fig. 1c), and expressed *THY1* gene at high level, which was a mesenchymal cell marker and was absent in DOX-iPSCs (Fig. 1d). However, when the DOX-iPS^diff^ cells were grown in LF2i medium plus Dox again, the cells could be reprogrammed and re-form AP-positive colonies, and showed the perfect iPS cell morphology after 2–3 passages (Fig. 1e). The re-formed DOX-iPS (DOX-iPS^2nd^) cells presented the high-level expression of transgenes and endogenous pluripotent genes, which was seen in DOX-iPSCs (Fig. 1a), but the expressions were significantly higher than that in DOX-iPS^diff^ cells (Fig. 1f). These results demonstrated that DOX-iPSCs could be used as a cell model to screen piPS cultural conditions since the differentiation state and pluripotent state of DOX-iPSCs were able to be switched depending on with or without the addition of Dox.

### Maintenance of DOX-iPSCs in 2i medium

**Fig. 2:**
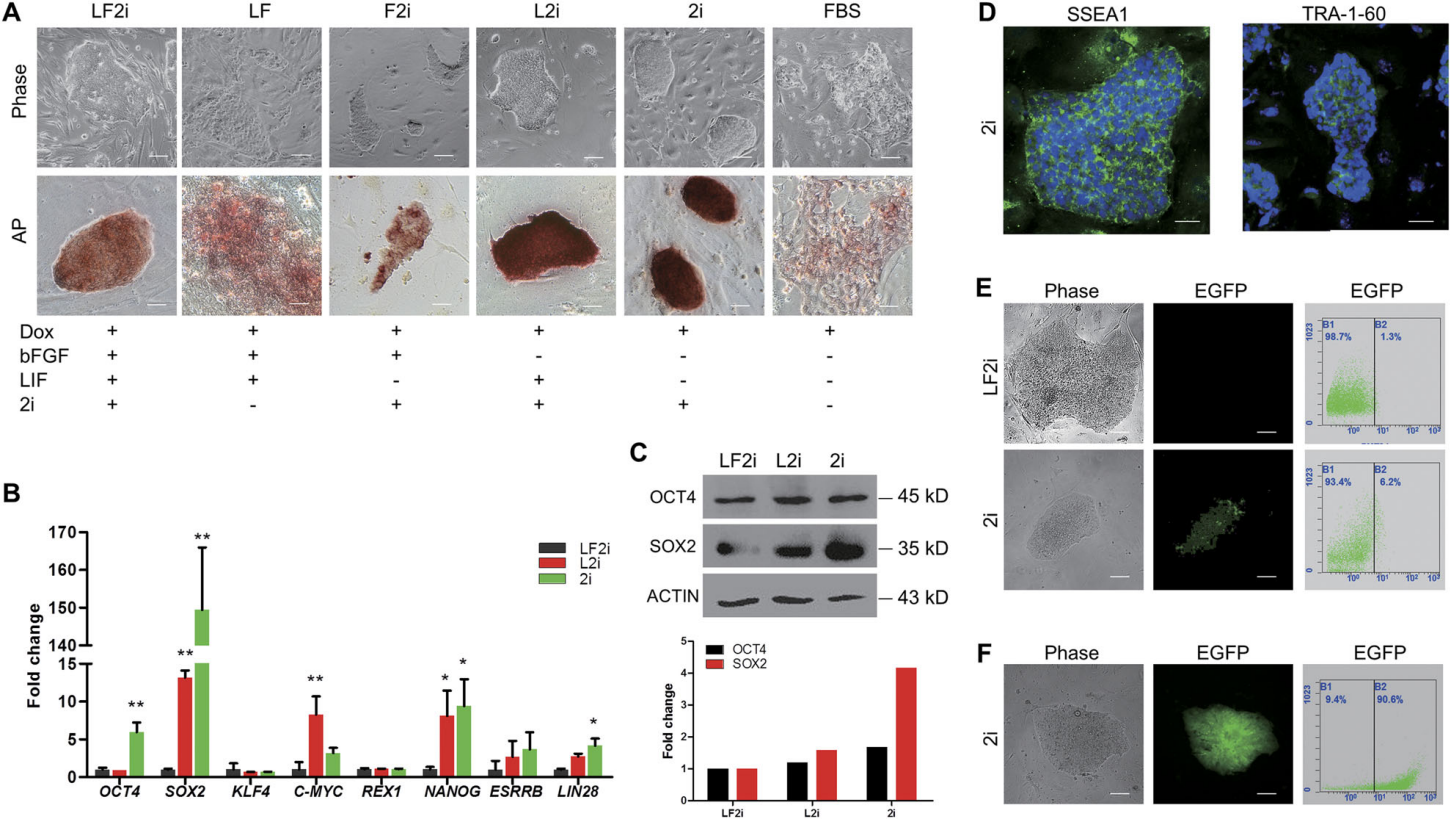
Maintenance of DOX-iPSCs in 2i medium and complete withdrawal of LIF and b-FGF cytokines. **a** DOX-iPSCs were cultured in media with or without LIF and b-FGF, and detected by alkaline phosphatase (AP) staining. Scale bar, 100 μm for phase and 50 μm for AP. **b** Quantitative RT-PCR analysis of endogenous pluripotent genes in DOX-iPSCs cultured in media with LF2i, L2i, and 2i, respectively. Data indicate mean ± SD. *P < 0.05, **P < 0.01, *n* = 3. **c** Western blotting and densitometry analysis of OCT4 and SOX2 expressions in DOX-iPSCs cultured in media with LF2i, L2i, and 2i, respectively. b-ACTIN was an internal control. **d** Immunofluorescence assay of SSEA1 and TRA-1-60 in DOX-iPSCs cultured in 2i medium. Scale bar, 50 μm. **e** Morphology and flow cytometry analysis of DOX-iPSCs that were infected by PL-SIN-EOS-S(4+)-EiP lentivirus and cultured in media with LF2i and 2i, respectively. Scale bar, 100 μm. **f** Morphology and flow cytometry analysis of PL-SIN-EOS-S(4+)-EiP infected DOX-iPS (DOX-iPS^eos^) cells that were cultured in 2i medium with puromycin selection. Scale bar, 100 μm

In our previous report^20^, we added LIF and b-FGF in the medium to culture and maintain piPSCs because both LIF and b-FGF are the essential cytokines for the maintenance of PSCs, in which mouse PSC required LIF signaling to maintain its naïve state and human PSC required b-FGF signaling to sustain its primed state^24^. To further identify the roles of LIF and b-FGF in porcine PSCs, we did the following screen assays by removing the individual cytokines and small molecules from LF2i medium, and then monitored the variations in morphology and self-renewal of DOX-iPSCs. In LF (-2i) and F2i (-LIF) media, colonies of DOX-iPSCs became flattened and incompact with ragged and uneven edges, and the AP staining was heavily reduced versus the DOX-iPSCs in LF2i medium (Fig. 2a). However, in the medium in which the two cytokines and small molecules were removed and only conserved Dox and fetal bovine serum (FBS), the colonies were completely differentiated, showing epithelial-like cell type. On the other hand, in the L2i medium in which b-FGF was removed, the colonies became more compact with a strong AP activity. Interestingly, we found that when DOX-iPSCs were cultured in 2i (-LIF, -b-FGF) medium, the colonies converted to the dome-shaped morphology with solid AP staining (Fig. 2a). The results of pluripotent gene expression showed that the endogenous pluripotent genes *OCT4*, *SOX2*, *NANOG*, and *LIN28* were significantly activated in 2i medium versus LF2i medium, and *c-MYC* was significantly activated in L2i medium versus LF2i and 2i media (Fig. 2b). *KLF4* expression was not influenced by altering the culture media, indicating that KLF4 might not be a key factor for porcine iPSCs as in our previous report^20^. However, other two vital pluripotent genes *REX1* and *ESRRB* were not activated in 2i medium yet. Western blotting analysis further confirmed the increased expression levels of OCT4 and SOX2 in 2i versus LF2i (Fig. 2c). DOX-iPSCs cultured in 2i medium also exhibited the positive immunostaining reaction of TRA-1–60 and SSEA1 (Fig. 2d). To further evaluate DOX-iPSCs in 2i medium, cells (DOX-iPS^eos^) infected by PL-SIN-EOS-S(4+)-EiP lentivirus, which has an EGFP reporter with SOX2 enhancer and permits non-invasive monitoring of SOX2 activity^36,37^, were cultured in LF2i and 2i media with puromycin selection. The flow cytometry analysis showed that there was a low percentage rate of DOX-iPS^eos^ cells in LF2i medium, conversely, 6.2% of the cells in 2i medium had SOX2 activity (Fig. 2e). The percentage rate of DOX-iPS^eos^ cells was largely enriched when the puromycin selection was conducted for 2–3 passages, which was able to efficiently eliminate the differentiated cells from the culture (Fig. 2f). These results indicated that the two key elements of Dox, which induced ectopic expression of four transcription factors OSKM, and 2i medium were able to maintain self-renewal of DOX-iPSCs without the addition of LIF and b-FGF cytokines. Also, DOX-iPSCs could be passaged by single-cell dissociation for a long term in vitro and maintained the dome-shaped morphology all along in the 2i medium. However, the morphology of DOX-iPSCs became flattened and incompact with ragged edges when the cells were cultured in LF2i medium for a long term (Supplementary Fig. 2).

### Reprogramming piPSCs in LF2i and 2i media

**Fig. 3:**
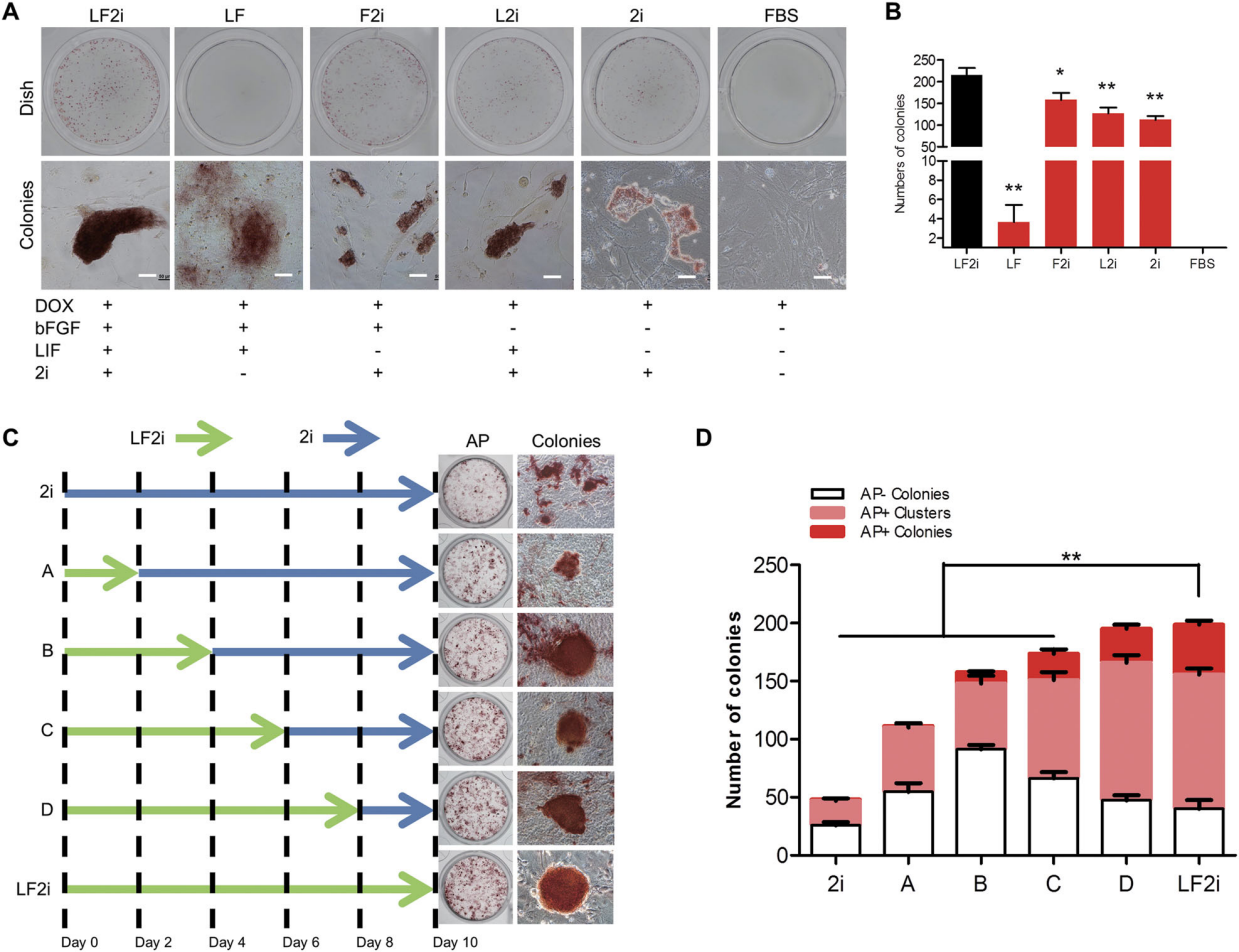
Reprogramming of porcine iPSCs in LF2i and 2i media. **a** DOX-iPS^diff^ cells were reprogrammed in media with or without 2i, LIF, and b-FGF for 4 days, and followed by alkaline phosphatase (AP) staining. Scale bar, 50 μm. **b** Counts of AP-positive colonies of DOX-iPS^2nd^ cells cultured in different media. **c** Reprogramming of PEFs into iPSCs by hOSKM in LF2i and 2i media for 10 days, and followed by AP staining. **d** Counts of AP-negative colonies, AP-positive clusters, and AP-positive colonies. Data indicate mean ± SD. *P < 0.05, **P < 0.01, *n* = 3

Although the 2i medium with Dox could maintain self-renewal of DOX-iPSCs, the question was that if the 2i medium could be used for cell reprogramming and to generate piPS cell lines. To answer this question, several culture conditions were applied to effectively reprogram DOX-iPS^diff^ cells into DOX-iPS^2nd^ cells. In LF and FBS media that do not have 2i, DOX-iPS^diff^ cells were unable to be completely reprogrammed into DOX-iPS^2nd^ cells, especially there were no AP-positive colonies in the FBS medium. In 2i medium, DOX-iPS^diff^ cells were also not fully reprogrammed since the morphology of the colonies was incompact and the AP activity was low (Fig. 3a, b). DOX-iPS^diff^ cells cultured in L2i and F2i media could form AP+ colonies; however, the size of the colonies was much smaller and less compact than the colonies seen in the LF2i medium. Additionally, the number of colonies in L2i and F2i media were also significantly reduced compared with that in the LF2i medium (Fig. 3b). This observation indicated that LF2i medium, but not 2i medium, was able to efficiently reprogram DOX-iPS^diff^ cells to DOX-iPS^2nd^ cells.

We then designed the following experiments to induce porcine somatic cell reprogramming in either LF2i or 2i medium or both media. Six induction approaches were set, which included an LF2i medium only induction, a 2i medium only induction, and four inductions using LF2i medium first for 2–8 days, and then changing to 2i medium for the rest of the induction term (Fig. 3c). The results of AP staining showed that the reprogrammed iPSCs were incomplete in 2i medium versus in LF2i medium; however, the cells could be finely reprogrammed when they were grown in the LF2i medium for 6–8 days, and then transferred to 2i medium though the number of AP+ colonies were still lower than that in the LF2i medium (Fig. 3d). This result suggested that the initiation of porcine cell reprogramming required LIF and b-FGF signaling pathways to start the endogenous pluripotent regulation network. As soon as the network was established, 2i condition could be functioned to maintain the self-renewal of the reprogrammed cells.

We also tried to grow DOX-iPSCs in Dox-free 2i medium. The concentration of Dox in 2i medium was reduced gradually from 4 µg/mL to 0 µg/mL. At the concentration of 1.0 µg/mL Dox, DOX-iPSCs retained the typical iPS cell morphology and high AP activity. However, when the concentration of Dox was reduced to 0.5 µg/mL, the cells altered the morphology, lost the AP activity, and started to differentiate (Supplementary Fig. 3A). The real-time polymerase chain reaction (RT-PCR) analysis further confirmed that when reduction of Dox to 0.5 µg/mL was carried out, the expression of transgenes and endogenous pluripotent genes significantly declined (Supplementary Fig. 3B-C), which indicated that 1.0 µg/mL Dox was the minimum concentration to maintain self-renewal of DOX-iPSCs, and Dox-free 2i medium cannot be applied to preserve DOX-iPSCs yet.

### Screening cytokines as a supplement for 2i medium

**Fig. 4:**
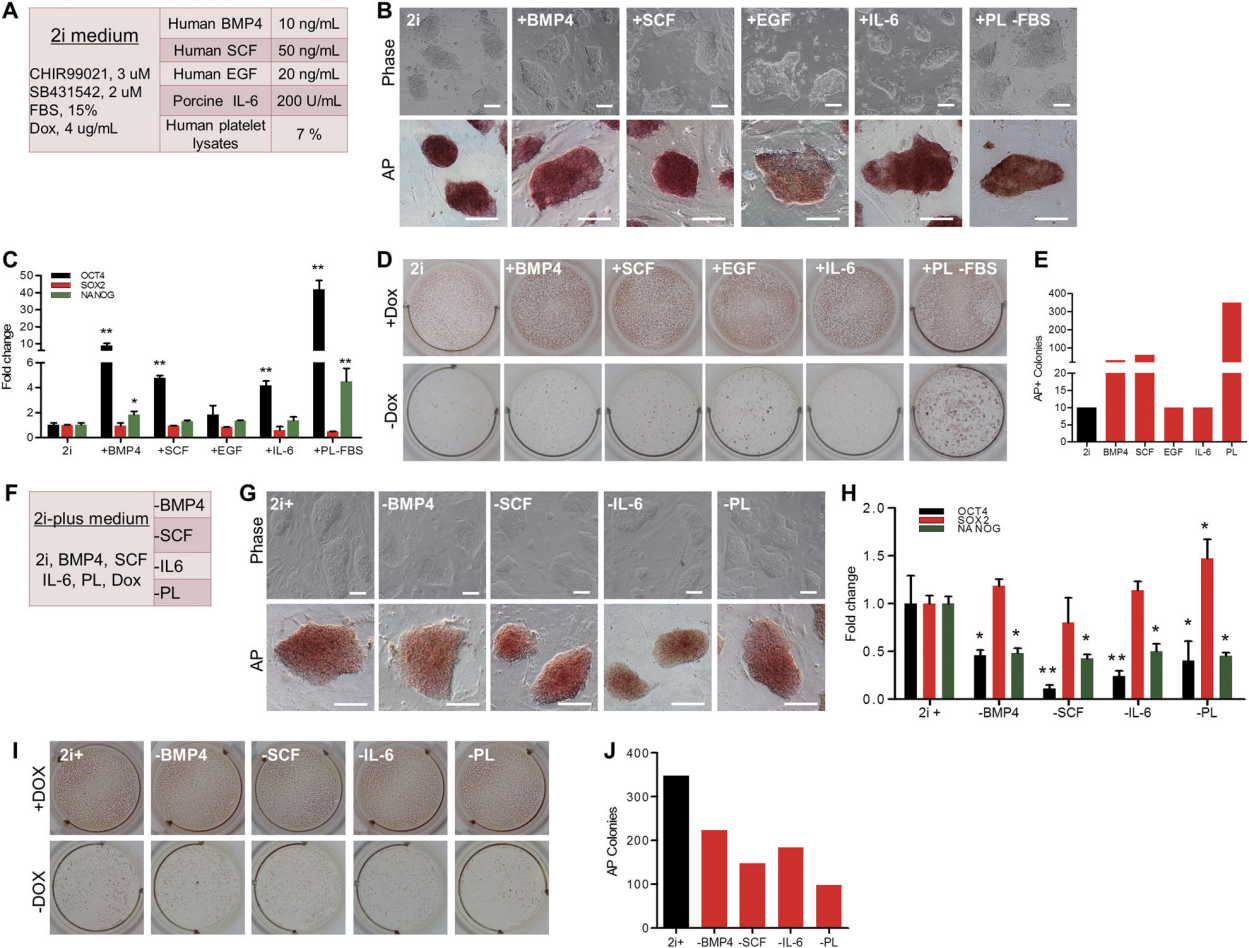
Substitution of FBS in 2i medium with different cytokines. **a** Ingredients of 2i medium and the supplementary cytokines. **b** DOX-iPSCs were cultured in 2i medium supplemented with an individual cytokine. Scale bar, 100 μm. **c** Quantitative RT-PCR analysis of pluripotent genes in DOX-iPSCs from **b** experiments. Growth of DOX-iPSCs in 2i medium supplemented with an individual cytokine and with (+Dox) or without (−Dox) doxycycline **d**, and counts of alkaline phosphatase (AP)-positive colonies from −Dox treatment **e**. **f** Ingredients of 2i-plus medium. **g** DOX-iPSCs were cultured in 2i-plus medium (2i+) by removing an individual cytokine. Scale bar, 100 μm. **h** Quantitative RT-PCR analysis of endogenous pluripotent genes in DOX-iPSCs from **g** experiments. Growth of DOX-iPSCs in 2i-plus medium by removing an individual cytokine and with (+Dox) or without (−Dox) doxycycline **i**, and counts of AP-positive colonies from −Dox treatment **j**. Data indicate mean ± SD. *P < 0.05, **P < 0.01, *n* = 3

There are many cytokines being used to sustain self-renewal and pluripotency in human and mouse PSCs^24^. To optimize the 2i medium, we selected five cytokines to replace FBS in the 2i medium (Fig. 4a). In the previous experiments, we realized that 2i medium without the addition of Dox was insufficient to maintain the self-renewal of DOX-iPSCs (Fig. 3). To enrich the ingredients of the 2i medium and to substitute FBS, we added individual cytokines into the 2i medium and found that addition of BMP4, SCF, and IL-6 in the 2i medium did not evidently influence the morphology and AP activity of DOX-iPSCs, but addition of EGF did cause morphology change and reduced AP activity (Fig. 4b). Gene expression assay indicated that adding individual BMP4, SCF, and IL-6 in the 2i medium could significantly elevate endogenous *OCT4* expression and slightly increase *NANOG* expression, but did not affect *SOX2* expression (Fig. 4c). Interestingly, when FBS was replaced with human platelet lysates (PL) in the 2i medium, the DOX-iPSCs morphology and AP activity remained in good shape as DOX-iPSCs grown in the 2i medium and the expression of endogenous *OCT4* and *NANOG* significantly increased (Fig. 4b, c). Next, we tested to withdraw Dox from the 2i medium and to evaluate the growth of DOX-iPSCs. Overall, removing Dox from the 2i medium could significantly reduce the number of AP+ colonies (Fig. 4d). However, addition of BMP4 and SCF in the Dox-free 2i medium could slightly increase the number of AP+ colonies versus the control, and addition of PL in the Dox-free/FBS-free 2i medium could prominently increase the number of AP+ colonies (Fig. 4d, e). This result indicated that the cytokines BMP4 and SCF and serum substitute PL could be used to maintain DOX-iPSCs subsisting on serum-free and Dox-free 2i condition. Additionally, though the addition of IL-6 in Dox-free 2i medium did not evidently increase the number of AP+ colonies, IL-6 could significantly increase the endogenous *OCT4* expression (Fig. 4c). Thus, we made a transitional serum-free 2i-plus medium that consisted of 2i, BMP4, SCF, IL-6, PL, and Dox (Fig. 4f). In the 2i-plus medium, DOX-iPSCs could sustain the self-renewal in vitro for a long term. When withdrawing individual components from the 2i-plus medium, in the first 2–3 passages, the morphology and AP activity of DOX-iPSCs remained the same (Fig. 4g); however, the gene expression profiles were significantly altered. Removing BMP4, SCF, IL-6, and PL individually from 2i-plus medium, the expressions of *OCT4* and *NANOG* were significantly downregulated (Fig. 4h). We also tried to grow DOX-iPSCs in Dox-free 2i-plus medium and found that the number of AP-positive colonies were extensively reduced in 2i-plus-1 medium versus the 2i-plus medium (Fig. 4i–j), indicating that all the components in the 2i-plus medium were important and played the basic role to maintain DOX-iPSCs growth. However, we noticed that without the addition of Dox in the 2i-plus medium, the pluripotent state of DOX-iPSCs could only be maintained for several passages. Thus, further optimization of the 2i-plus medium is required.

### Screening small molecules as a supplement for 2i-plus medium

**Fig. 5:**
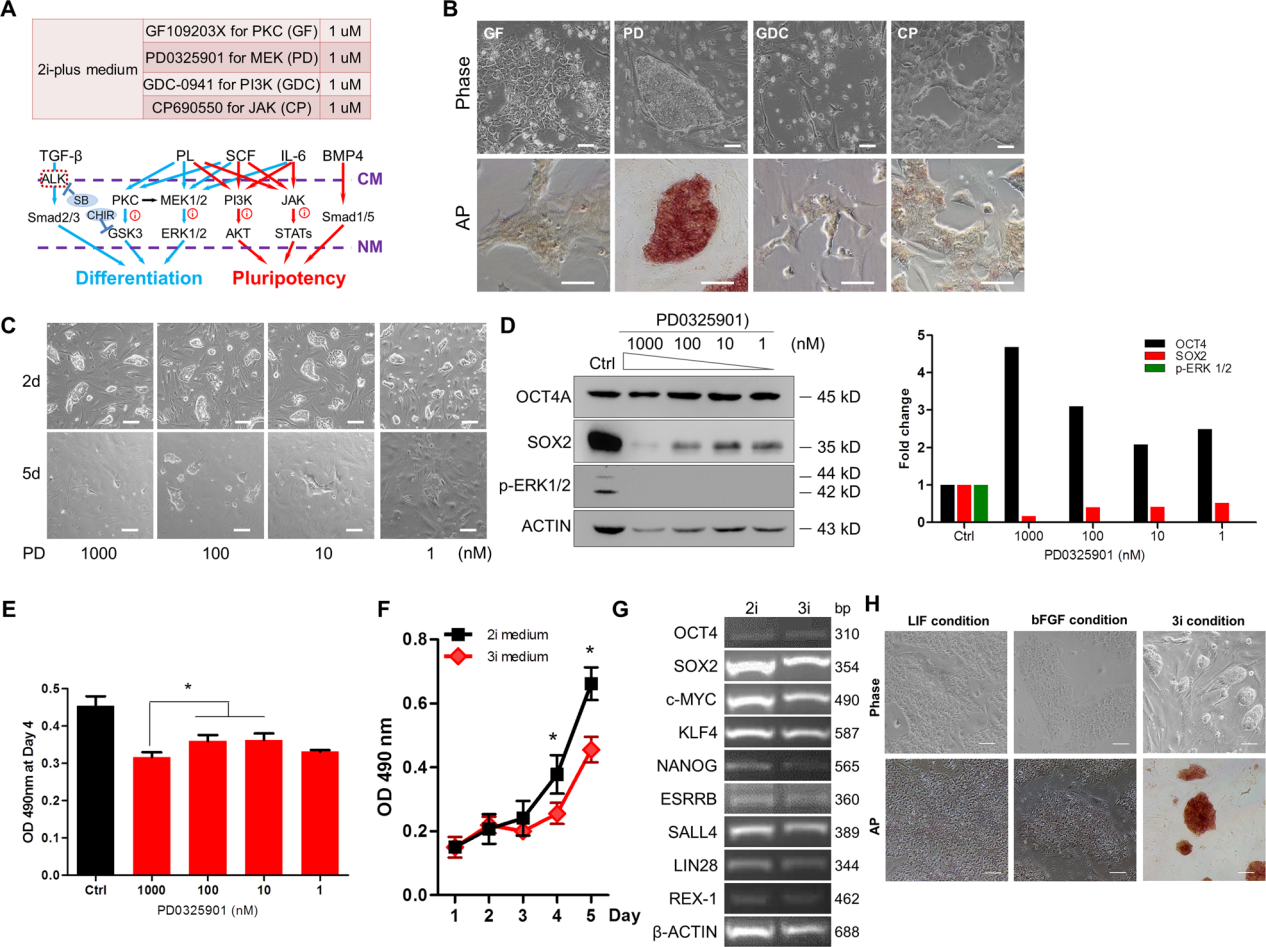
Substitution of doxycycline in 2i-plus medium with small molecules. **a** Ingredients of 2i-plus medium and the diagram of signaling networks that cytokines and inhibitors are involved. **b** DOX-iPSCs were cultured in 2i-plus medium supplemented with small molecules. **c** DOX-iPSCs growth in Dox-free 2i-plus medium supplementary with 1–1000 nM PD0325901 (PD) for 2 and 5 days. **d** Western blot and densitometry analysis of OCT4, SOX2, and phosphorylated ERK in DOX-iPSCs cultured in Dox-free 2i-plus medium with PD. b-ACTIN was an internal control. **e** MTT assay of DOX-iPSCs growth in Dox-free 2i-plus medium with PD for 4 days. **f** Growth curve of DOX-iPSCs in 2i and 3i (2i-plus medium without Dox and with 500 nM PD) media. **g** RT-PCR analysis of pluripotent genes in DOX-iPSCs cultured in 2i and 3i media. **h** Morphology and alkaline phosphatase staining of DOX-iPSCs cultured in LIF-dependent and b-FGF-dependent media without the addition of Dox, and 3i medium. Scale bar, 100 μm. Data indicate mean ± SD. *P < 0.05, *n* = 3

The components in the 2i-plus medium involve in several signaling pathways, which include PKC, MAPK, PI3K-AKT, JAK-STATs, and TGF-beta family, and regulate self-renewal and pluripotency of PSCs (Fig. 5a). To understand whether these pathways promote cell differentiation or pluripotency in piPSCs, we selected four small molecules that included GF109203X for PKC (PKCi), PD0325901 for MEK (MEKi), GDC-0941 for PI3K (PI3Ki), and CP690550 for JAK (JAKi) (Fig. 5a). Addition of PKCi, PI3Ki, and JAKi into the 2i-plus medium led to cell differentiation and loss of AP staining, indicating that these pathways are crucial for the self-renewal of DOX-iPSCs (Fig. 5b). Alternatively, the inhibition of MEK pathway by PD0325901 (PD) did not affect the cell morphology and AP activity, but PD did cause the cells grow more slowly. Previous report proved that the phosphorylation cascade (RAS/RAF/MEK/ERK) of MAPK pathway controlled the cellular proliferation and differentiation^32,38,39^, which could explain that the inhibition of MEK pathway sustained the undifferentiated state and arrested the cell growth. Next, a serial concentration of PD (1–1000 nM) was applied in Dox-free 2i-plus medium. When PD concentration was 100 nM or less, cell growth arrest was relieved; however, cell differentiation started (Fig. 5c). On the other hand, cell morphology and growth rate could be retained as normal DOX-iPSCs when PD concentration was between 200 and 600 nM (Supplementary Fig. 4). Western blotting confirmed that the addition of PD completely abolished the phosphorylated ERK1/2, and elevated endogenous OCT4 level (Fig. 5d). Unexpectedly, we noticed that SOX2 level was reduced, showing the negative correlation with PD concentration, which is worth to further investigate the regulatory mechanism. The viability of DOX-iPSCs in the Dox-free 2i-plus medium with PD was detected, and the results showed that high level of PD significantly blocked the cell growth (Fig. 5e). Therefore, we found that 0.5 μM PD was sufficient for the maintenance of pluripotency and without serious blocking of cell growth (Supplementary Fig. 4). At this point, we converted 2i-plus medium to 3i medium by removing Dox and adding 0.5 μM PD.

The growth rate of DOX-iPSCs was slower in 3i medium compared to 2i medium (Fig. 5f). However, pluripotent gene expression profiles did not change in 3i medium (Fig. 5g). This result indicated that in 3i condition, transgenes in DOX-iPSCs were completely switched off due to the withdrawal of Dox, and endogenous pluripotent genes were activated and retained the self-renewal of DOX-iPSCs. Next, we cultured DOX-iPSCs in 3i medium paralleled with LIF-dependent culture condition^17^, and b-FGF-dependent culture condition^14^. The result showed that DOX-iPSCs survived only in the 3i medium, in which the cells showed the typical iPSC morphology and strong AP activity, but DOX-iPSCs could not survive in both LIF-dependent and b-FGF-dependent media (Fig. 5h).

### Culture of different piPS cell lines in 3i medium

**Fig. 6:**
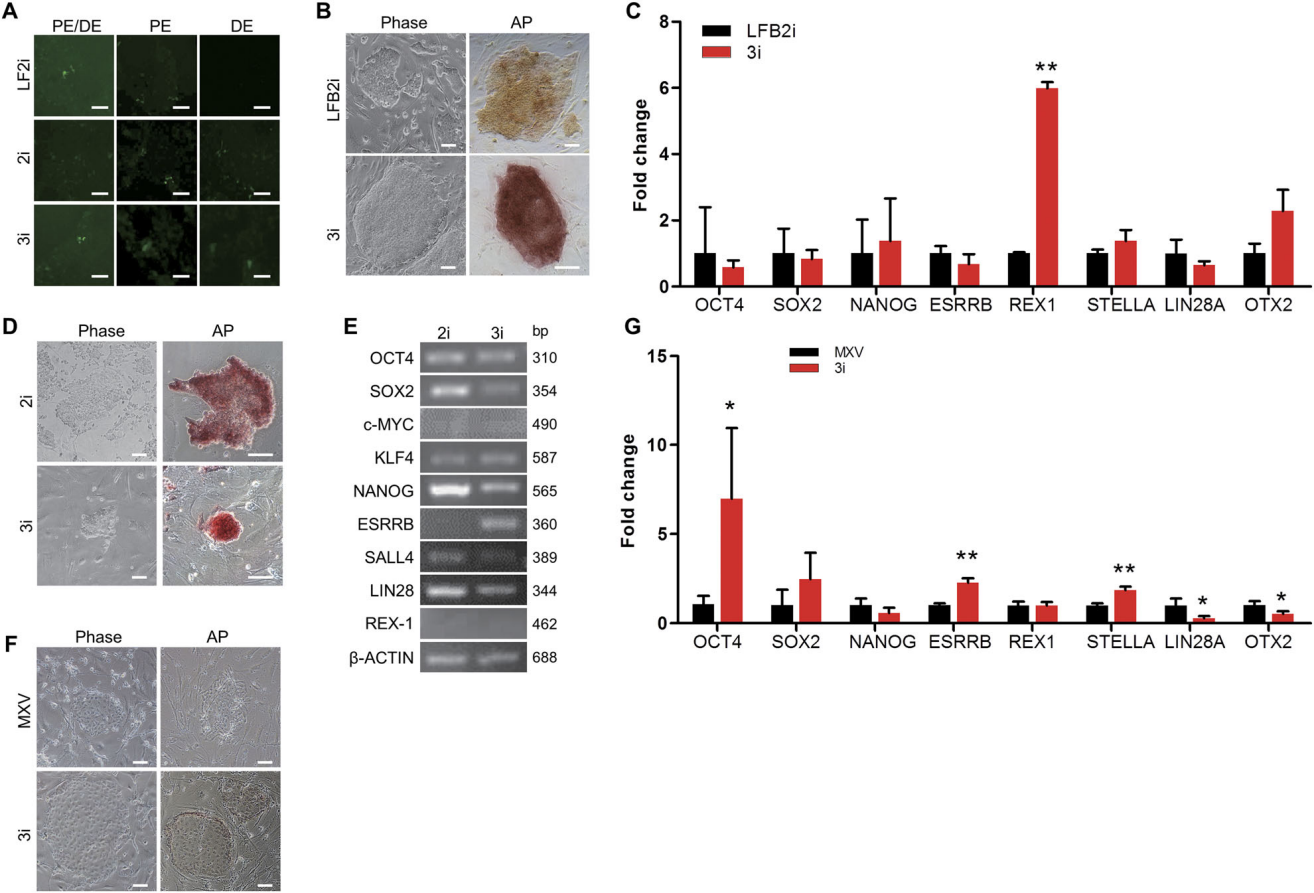
Maintenance of porcine iPS cell lines in 3i medium. **a** Fluorescence analysis of *OCT4* promoter (PE/DE), *OCT4* proximal enhancer (PE), and distal enhancer (DE) in DOX-iPSCs with different media. **b** Morphology and alkaline phosphatase (AP) staining of LFB2i-piPSCs grown in LFB2i and 3i media. **c** Quantitative RT-PCR analysis of pluripotent genes from LFB2i-piPSCs in LFB2i and 3i media. **d** Morphology and AP staining of iPF4-2 cells grown in 2i and 3i media. **e** RT-PCR analysis of pluripotent genes from iPF4-2 cells grown in 2i and 3i media. **f** Morphology and AP staining of pPSCs grown in MXV and 3i media. **g** Quantitative RT-PCR analysis of pluripotent genes from pPSCs in MXV and 3i media. Scale bar, 100 μm. Data indicate mean ± SD. *P < 0.05, **P < 0.01, *n* = 3

In order to verify the pluripotent state of DOX-iPSCs grown in LF2i, 2i, and 3i conditions, three *OCT4* promoter reporters, which included the full-length *OCT4* promoter (PE/DE), proximal enhancer (PE), and distal enhancer (DE) (Supplementary Fig. 5), were transfected into DOX-iPSCs. In LF2i condition, full length of *OCT4* promoter and proximal enhancer were activated, but distal enhancer was inactive, indicating that DOX-iPSCs cultured in LF2i medium were in the primed-like state. Alternatively, in 2i and 3i conditions, not only the full length of *OCT4* promoter and distal enhancer were activated, but the proximal enhancer was also activated, indicating that DOX-iPSCs cultured in 2i and 3i conditions were in the naïve-like state (Fig. 6a).

To further verify whether the 3i medium was extensively able to maintain self-renewal of porcine PSCs, two porcine iPS cell lines were cultured in the 3i medium. The LFB2i-piPS, which was an intermediate state piPS cell line and showed loose morphology and low AP activity, was cultured in LFB2i medium^20^. When LFB2i-piPSCs were transferred from LFB2i medium to 3i medium, a large number of cells died on the first 2 days. Continuing the culture of LFB2i-piPSCs in 3i medium for 4–6 days, the cells started to grow and form typical iPSC colonies, which showed compact morphology and high AP activity (Fig. 6b). Quantitative RT-PCR assay showed that the expression level of *REX1* in LFB2i-piPSCs was significantly increased in 3i condition (Fig. 6c). Expression of several other pluripotent genes, including *NANOG*, *STELLA*, and *OTX2*, was also slightly elevated, but was not statistical significant. The second porcine iPS cell line cultured in 3i medium was iPF4-2 (Supplementary Fig. 6), which is a doxycycline-inducible porcine iPS cell line reported by Xiao’s laboratory^13^. When iPF4-2 cells grown in the reported medium were transferred directly in 3i medium, the cells stopped growing. Therefore, we used the following strategy. The cells were firstly cultured in 2i medium with 10 μg/mL Dox for 3 passages. The morphology and AP activity of iPF4-2 are seen in Fig. 6d. The cells were then transferred to 3i medium and continued culturing for 2 passages. The iPF4-2 cells formed small and compact domed colonies with high AP activity in 3i medium (Fig. 6d). Results of RT-PCR analysis showed that the expression of pluripotent genes of iPF4-2 cells was very similar between 2i and 3i media, but *ESRRB* expression was obviously increased in 3i condition (Fig. 6e). Interestingly, the expression of *c-MYC*, which was one of the transgenes and should be inducible by Dox in 2i medium, was detected to be at very low level in both 2i and 3i media. Also, *REX-1* was not detectable in both 2i and 3i media due to the unclear feature of this cell line. Furthermore, a porcine pluripotent stem cell (pPSC) line, which was reported by Liu’s laboratory^40^, grown in MXV medium, and showed the flattened and loose morphology, was cultured in 3i medium. Since pPSCs showed the feature of trophoblastic stem cell morphology and low AP activity, the cells cultured in 3i medium did not present the distinct phenotypic changes (Fig. 6f); however, the expression of endogenous pluripotent genes, including *OCT4*, *ESRRB*, and *STELLA*, was significantly increased. On the other hand, the expression of *LIN28* and *OTX2*, which were the primed state markers, was significantly decreased (Fig. 6g). Overall, 3i medium can be used to maintain porcine PSCs derived from different culture conditions. It is worth to further investigate whether PSCs cultured in 3i medium retained the potential to generate the chimera embryos and chimera piglets.

## Discussion

The potentials of porcine PSCs promised in transgenic cloned pigs and allogenic organ preparation have prompted widespread interests. However, obtaining authentic porcine PSCs is still a big challenge. At the starting point, we considered that the optimization of the culture conditions for preserving porcine PSCs was greatly facilitated by establishing ectopic controllable piPSCs. In mouse, Dox-inducible lentiviral vectors that carried pluripotent transcription factors were able to reprogram mouse embryonic fibroblasts (MEFs) into stable iPSCs that maintained the pluripotency in standard mESC culture condition after Dox withdrawal^41^. Taking a similar strategy, Dox-induced *KLF2* and *NANOG* vectors also facilitated the identification of putative naïve human pluripotency culture condition^29^. The previous study had reported Dox-induced porcine iPSCs that showed the typical primed state iPSC morphology under the culture condition with Dox^13^. In this study, we generated DOX-iPSCs using lentiviral particles packaged with TetO-FUW-OSKM and FUW-M2rtTA vectors, which were also used for reprogramming mouse and human somatic cells into iPSCs^42^. DOX-iPSCs cultured in LF2i medium relied on the addition of Dox and presented a flattened morphology. As soon as Dox was withdrawn, the expression of transgenes completely vanished, and DOX-iPSCs started to differentiate, presenting fibroblastoid morphology and loss of AP activity. However, when DOX-iPS^diff^ cells were grown in the medium with Dox again, the cells were reprogrammed and re-formed AP-positive colonies with the perfect iPSC morphology, demonstrating that DOX-iPSCs were able to be switched between the pluripotent state and differentiation state depending on with or without addition of Dox. Thus, this is a useful cell model to screen the cultural conditions for maintaining self-renewal and pluripotency of piPSCs, when completely turns off the expression of transgenes in the cells.

Our results showed that the balance of specific stage of pluripotency established based on Dox and LF2i was easy to be broken once Dox was withdrawn, meaning shut down of the expression of exogenous transcription factors. These observations indicated that the LF2i condition only was insufficient for the maintenance of piPSCs. Previous studies showed that the application of LIF or b-FGF was essential for the maintenance of porcine pluripotency^14^. Actually, collective application of LIF and b-FGF in the medium for culturing piPSCs was able to accelerate the reprogramming processes^20^. The usage of LIF or b-FGF in porcine studies was based on the naïve state mouse PSCs that required LIF to activate the JAK-STAT3 pathway, and the primed state human PSCs that required b-FGF/Activin A pathways^24^. However, the previous reports showed that the signaling pathways used for maintaining human and mouse PSCs might not sustain the self-renewal and pluripotency of porcine PSCs^21,22^. To meet the fundamental need for the maintenance of DOX-iPSCs, alternative cytokine screening to replace LF2i medium was performed. Surprisingly, when LIF and b-FGF were removed from the culture condition simultaneously, 2i plus Dox was sufficient not only for the maintenance of DOX-iPSCs self-renewal, but also for elevating the expression of endogenous pluripotent genes *OCT4*, *SOX2*, and *NANOG*. However, the culture condition of 2i medium with Dox failed to reprogram DOX-iPS^diff^ cells versus LF2i condition, indicating that the porcine somatic cell reprogramming and PSCs maintaining might need different regulatory networks. We noticed that further withdrawal of Dox from the 2i medium caused DOX-iPSCs differentiation, indicating that porcine endogenous pluripotent genes could not fully activate under the serum only condition. Thus, in 2i-plus medium, the serum was replaced with PL, and cytokines LIF and b-FGF were replaced by BMP4, SCF, and IL-6. The results demonstrated that 2i-plus medium could diminish the differentiation of DOX-iPSCs when Dox was withdrawn. The bypass of LIF and b-FGF signaling pathways would be conducive to activation of other regulatory networks that contribute to maintaining pluripotency of porcine PSCs.

Two small molecule inhibitors (2i) CHIR99021 (for GSK3β inhibitor) and PD (for MEK inhibitor) were sufficient to maintain mouse naïve pluripotency without LIF supplement^32^. In human naïve pluripotency, the inhibition of these two pathways was also proved important^24,28–30^. However, in pig, supplement of 2i at the early stage of porcine cell reprogramming resulted in cell growth arrest^43^. We also tried to add MEK inhibitor PD into the LF2i medium, and found that supplement of PD resulted in massive cell growth arrest (data not shown). We then tried to add PD in 2i-plus medium with lower concentration, and found that PD showed irreplaceable effect in the maintenance of porcine pluripotency (Fig. 5). These results indicated that the application of PD in porcine pluripotency maintenance should be in an appropriate concentration.

PL retain abundant growth factors and cytokines that are stored in platelet granules and are able to be released by freeze/thaw-mediated lysis^44^. The PL have been used to culture regenerative cells such as mesenchymal stem cells and progenitor cells^44,45^. In the pig, the previous reports showed that iPSC could be derived in serum-free systems^46^. In this study, we set up a 3i medium, in which the serum was replaced with PL, and showed that PL could promote the expression of endogenous pluripotent genes and increase the number of AP-positive colonies. These results indicated that the factors within PL could stimulate the self-renewal and pluripotency of porcine PSCs.

The previous studies in mouse and human PSCs showed that when cells were in the primed state, the *Oct4* PE was activated, and when cells were in the naïve state, the DE was activated^47^. We made porcine *OCT4* promoter reporters that retained the PE and DE elements, and applied to monitor the pluripotent states of porcine PSCs. We found that only PE was activated when DOX-iPSCs were in LF2i medium, whereas both PE and DE were activated when DOX-iPSCs were in 2i and 3i media, indicating that the cells grown in 2i and 3i media were closer to naïve-like state. Recent report showed that if mouse ESCs were cultured in naïve medium with FBS or KSR, both PE and DE enhancers were activated; however, if with N2B27, only DE was activated. Additionally, DNA methylation and histone modification also affected the heterogeneous activation of *Oct4* DE and PE enhancers^47^. In our experiments, we also found the heterogeneous activation of *OCT4* enhancers in DOX-iPSCs, indicating that the factors of medium supplements, DNA methylation, and histone modification might affect the naïve-state porcine PSCs.

Though our data provide new resolution for culturing porcine PSCs, there are several issues that need to be addressed in the future. First, though the serum-free 3i condition is able to apply for converting primed-like state porcine PSCs to naïve-like state cells, whether 3i condition can be used for two-step porcine iPS induction approach to gain the authentic pluripotency? Second, porcine PSCs grown in 3i condition showed slow proliferation compared to DOX-iPSCs grown in 2i condition. It will be interesting to find out the factors that lead to blocking of cell proliferation. Third, the golden criterion to prove the pluripotency is to generate germline-competent chimeras by injection of porcine PSCs into porcine post-implantation embryos. We do not know if PSCs grown in 3i medium retain the embryonic chimerism yet.

## Materials and methods

### Cell cultures

PEFs, which were prepared and reported previously in this laboratory^48^, were cultured in DMEM (Hyclone, USA) supplemented with 15% FBS (S601S-500, SeraPro, Germany), 0.1 mM non-essential amino acids (NEAA 11140–050, Gibico, USA), 1 mM L-glutaMAX (3505006, Gibico), and 50 units/50 mg/mL penicillin/streptomycin at 37 °C, 5% CO_2_ in a humidified atmosphere. HEK 293T and MEFs were cultured in DMEM supplemented with 10% FBS and 50 units/50 mg/mL penicillin/streptomycin. Porcine DOX-iPSCs were cultured in LF2i medium, consisting of DMEM supplemented with 15% FBS, 0.1 mM NEAA, 1 mM L-glutaMAX, 10 ng/mL LIF (LIF1050, Merck Millipore, USA), 10 ng/mL b-FGF (100-18B, PeproTech, USA), 0.1 mM β-mercaptoethanol (M3148, Sigma Aldrich, USA), 3 µM CHIR99021 (CHIR-50, StemRD, USA), 2 µM SB431542 (SB-50, StemRD), 4 µg/mL Dox (D9891, Sigma Aldrich), and 50 units/50 mg/mL penicillin/streptomycin. The 0.25% Trypsin (Invitrogen) was used to passage DOX-iPSCs every 3 days. Porcine intermediate iPS cell line LFB2i-piPSC, which was generated in this laboratory, was cultured in LFB2i medium^20^. The doxycycline inducible porcine iPS cell line iPF4-2, which was from Dr. Xiao’s laboratory^13^, was cultured in 2i medium supplemented with 10 µg/mL Dox. The porcine PSC line (pPSC), which was generated from 5.5 days blastocysts and was from Dr. Liu’s laboratory, was cultured in MXV medium under 5% oxygen atmosphere^40^. Porcine LFB2i-piPSC, pPSCs, and iPF4-2 cells grown in 3i medium were passaged by the manual approach. The 2i medium contains DMEM supplemented with 15% FBS, 0.1 mM NEAA, 1 mM L-glutaMAX, 3 µM CHIR99021, 2 µM SB431542, and 4 µg/mL Dox. The 2i-plus medium contains DMEM/F12 supplemented with 7% PL (SuMSC-SA, Sunny Biotechnology, USA), 0.1 mM NEAA, 1 mM L-glutaMAX, 200 U/mL IL-6 (PSC0064. Thermo Fisher), 10 ng/mL BMP4, 50 ng/mL SCF (300–07, PeproTech), 3 µM CHIR99021, 2 µM SB431542, and 4 µg/mL Dox. Four small molecules added into 2i-plus medium were GF109203X (A8342, APE Biotechnology, China), PD0325901 (PD-50, StemRD), GDC-0941 (A8210, APE Biotechnology), and CP690550 (A4135, APE Biotechnology), respectively. The 3i medium contains DMEM/F12 supplemented with 7% PL, 0.1 mM NEAA, 1 mM L-glutaMAX, 200 U/mL IL-6, 10 ng/mL BMP4, 50 ng/mL SCF, 3 µM CHIR99021, 2 µM SB431542, and 0.5 µM PD. The detailed formular of  culture mediums are listed in Supplementary Table 1.

### Generation of piPS cell lines

The 293T cells were planted on a 6-well plate with 2 × 10^6^ cells per well. After 24 h, lentiviral vectors TetO-FUW-OSKM and FUW-M2rtTA were transfected into 293T cells, respectively, by using TurboFect Transfection Reagent (R0531, Thermo Fisher) according to the manufacturer's instruction. At 48 h post transfection, the medium with viral particles was collected from each individual transfection and filtered through a 0.45-mm cellulose acetate filter (Millipore). An equal ratio of viral particles was mixed and used to infect PEF cells with 4 µg/mL polybrene for 12 h. To increase the infection efficiency, a second round of infection could be conducted for another 12 h. The infected cells were re-plated on a 60-mm dish (50,000 cells/plate) and cultured in LF2i medium with 4 µg/mL Dox for 6–8 days, and then iPS-like colonies appeared. The colonies were picked up, cut into pieces with a glass needle, and seeded on a plate coated by MEF feeders to continue the growth. After several passages, piPS colonies were collected and used to detect the expressions of transgenes and endogenous pluripotent genes.

To reprogram PEFs with retrovirus pMXs carrying human *OCT4*, *SOX2*, *KLF4*, and *c-MYC*, the virus particles were prepared as described previously^20^. The reprogrammed cells were cultured in either LF2i or 2i medium for 10 days. The lentiviral vector of PL-SIN-EOS-S(4+)-EiP was transfected into 293T cells to make virus particles. Lentiviral particles were infected into DOX-iPSCs following the procedure as described above, and the infected DOX-iPSCs were cultured with 1 µg/mL puromycin. Plasmid vectors used for lentiviral production are listed in Supplementary Table 2.

### AP staining

AP activity of piPSCs was detected by AST Fast Red TR and α-Naphthol AS-MX Phosphate (Sigma Aldrich) according to the manufacturer’s instructions. Briefly, cells were washed twice using ice-cold PBS, and fixed with 4% paraformaldehyde in PBS (pH 7.4) for 15 min at room temperature, and washed three times with ice-cold PBS. The cells were then incubated with the mixture (1.0 mg/mL Fast Red TR, 0.4 mg/mL Naphthol AS-MX in 0.1 M Tris Buffer) at room temperature. After 10 min incubation, the AP-positive iPS colonies showed in red color. The images were documented by a Nikon fluorescence microscope.

### Construction of porcine OCT4 promoter-based reporter vectors

To clone the porcine *OCT4* promoter, total genomic DNA was extracted from PEF cells. The 4.2 kb promoter fragment with 5′UTR sequence of *OCT4* gene was amplified by PCR using CloneAmp™ HiFi PCR Premix (#639298, Clontech, USA). PCR fragment was subcloned into pGEM-T Easy vector (A1360, Promega, USA) and confirmed by DNA sequencing. To construct reporter vectors, the full-length OCT4 promoter (4.2 kb), DE (3.1 kb), and PE (1.1 kb) were subcloned into pEGFP-1 vector, respectively. The three reporters were respectively transfected into piPS cells using Lipofectamine 3000 (L3000015, Thermo Fisher) according to the instruction. Fluorescent images were documented by an EVOS fluorescence microscope at 48 h post transfection. Primers used in vectors construction are listed in Supplementary Table 3, and all the constructed plasmid vectors are listed in Supplementary Table 2.

### Karyotype analysis

DOX-iPSCs were grown in a 60 mm culture dish with LF2i medium and 50 μg/mL demecolcine for 1 h. The cells were then trypsinized and harvested for karyotype analysis. The procedure of karyotype analysis was routinely performed as the previous description^19^.

### Flow cytometry

DOX-iPSCs infected by PL-SIN-EOS-S(4+)-EiP lentiviral were used for flow cytometry analysis. The cells were harvested at 72 h after infection, or after puromycin selection, the harvested cells were trypsinized into a single cell, washed two times with PBS, and resuspended in PBS. The cells were then analyzed by the flow cytometer (Beckman Coulter, Brea, CA) to sort GFP-positive cells. DOX-iPSCs with no fluorescence were used as a negative control.

### Immunocytochemistry

To perform immunocytochemistry analysis, the cells were fixed with 4% paraformaldehyde in PBS (pH 7.4) for 15 min at room temperature. Fixed cells were washed twice with ice-cold PBS and were subsequently incubated with PBS containing 0.1% Triton X-100 for 10 min, and washed again in PBS for three times. After blocking unspecific bindings with BSA-blotting buffer (1% BSA, 0.1% Tween 20 in PBS) for 30 min, the cells were incubated with BSA-blotting buffer and antibodies of anti-SSEA1 (4746s, Cell Signaling Technology, USA), anti-Tra-1–60 (4746s, Cell Signaling Technology), anti-OCT4 (SC-5279, Santa Cruz Biotechnology, USA), anti-NANOG (ab80892, Abcam, USA), and anti-SOX2 (3579s, Cell Signaling Technology) kept in a humidified chamber at 4 °C overnight or at 37 °C for 1 h. After washing with PBS for three times, the cells were incubated with FITC conjugated secondary antibody for 1 h at room temperature. The nuclei were stained by 10 µg/mL Hoechst 33342 for 2 min. The images were documented by an EVOS fluorescence microscope.

### RT-PCR

Total cellular RNA of piPS and PEF cells was extracted by TRIzol Reagent (Invitrogen) according to the manufacturer’s procedure. The quality of RNA samples was determined by the measurement of 260/280 ratio, and samples in a ratio of 2.0 were used for reverse transcription. The DNase I treatment was sometimes utilized in RNA samples to remove genomic DNA contamination. One microgram RNA was reverse transcribed with oligo-dT primer using RevertAid™ reverse transcriptase (Thermo Fisher). PCR reactions were performed for 32 cycles at 94 °C 30 s, 55 °C 30 s, and 72 °C 30 s. The negative control was done by directly performing PCR with total RNAs to check the genomic DNA contamination, and β-Actin was used as an internal control. Quantitative RT-PCR analyses were performed in triplicates using SYBR Green PCR Master Mix (Takara), and the data were normalized to β-Actin mRNA. Primer sequences are provided in Supplementary Table 3.

### Embryoid body formation and spontaneous differentiation

Porcine iPS cells were cultured in a 35 mm Petri dish through suspension culture (2 × 10^6^ cells/plate) in 2i medium without Dox. The culture medium was replaced every 2 days. After 5 days in suspension culture, the formed embryoid bodies (EBs) were transferred to a gelatin-coated culture dish allowing the spontaneous differentiation for another 5 days. The cells were then fixed and used for immunocytochemistry analysis to detect markers of the three germ layers, including TUJ1 (MMS-435P, Covance, USA) for ectoderm, DESMIN (MAB3430, Millipore) for mesoderm, and AFP (MAB1368, R&D System, USA) for endoderm.

### Western blotting

To determine the expressions of OCT4, SOX2, and phosphorylated Erk1/2, DOX-iPSCs were lysed by RIPA buffer (Thermo Scientific) for 10 min on ice, resuspended in 5× SDS-PAGE loading buffer (50 mM Tris-HCl pH 6.8, 2% SDS, 10% glycerol, 2% β-mercaptoethanol, and 0.05% bromophenol blue), and heated at 100 °C for 5 min. A 15-μL cell lysate was loaded onto 12% SDS-PAGE gel. After electrophoresis, the proteins were transferred to a PVDF membrane by semidry electrophoretic transfer (Bio-Rad) for 45 min at 15 V. The membrane was blocked with blocking buffer (20 mM Tris/HCl pH7.6, 137 mM NaCl, 0.1% Tween 20, and 8% skim milk) at 25 °C for 2 h, and then incubated with the anti-OCT4 antibody, anti-phosphorylated Erk1/2 antibody (4377s, Cell Signaling Technology), and anti-SOX2 antibody in the blocking buffer at 4 °C overnight, respectively. After washing three times with TBS-T buffer (20 mM Tris/HCl pH 7.6, 137 mM NaCl, 0.1% Tween 20), the membrane was incubated with secondary antibody at room temperature for 1 h. After washing three times in TBS-T, the membrane was incubated in the enhanced chemiluminescent substrate (#32106, Pierce) and detected with a Chemiluminescent Imaging System (ZY058176, Tanon-4200, China). The anti-beta-ACTIN (KM9001, Sungene Biotech, China) antibody was used as an internal control.

### MTT cell proliferation assay

Thiazolyl blue tetrazolium bromide (MTT, M5655, Sigma) powder was dissolved in PBS at the concentration of 0.5 mg/mL. After removing the culture medium, MTT was added into the culture dish, and incubated at 37 °C for 2 h. MTT was removed, and the same amount of DMSO (D2650, Sigma) was added into the dish for 1–2 min, and then DMSO was recovered and used for the O.D. reading by the microplate reader (ELx808, Gene Co., Ltd., Hong Kong, China) under the 490 nm wavelength.

### Statistical analysis

All the experiments were of three biological replicates, except for the experiments of cytokines screening as supplement ingredients for 2i medium which were replicated twice. Statistical analyses were performed with the two-way ANOVA that was used to study the differences between grouped data, and Student’s *t*-test was performed with one-way analysis. Statistical significance was accepted at *P* < 0.05.

## Electronic supplementary material

Supplementary figure legends
