# Supplementary material for: Preserving self-renewal of porcine pluripotent stem cells in serum-free 3i culture condition and independent of LIF and b-FGF cytokines
Source: Cell Death Discov. 2018 Feb 14;4:21. doi: 10.1038/s41420-017-0015-4 (PMC5841441; doi:10.1038/s41420-017-0015-4)
Supplement: Supplementary file 1 — Supplementary figure legends [file 41420_2017_15_MOESM1_ESM.pdf]

Supplemental figure legends

Figure S1. Characterization of porcine DOX-iPSCs. **A.** Morphology and alkaline phosphatase staining of PEF cells and three porcine DOX-iPS cell lines A1, B2, and D1. **B.** PCR analysis of transgenes and RT-PCR analysis of endogenous pluripotent genes of A1, B2, D1, and PEF cells. *b-Actin* was used as internal control. **C.** Immunofluorescence assays of OCT4, SOX2, NANOG, and SSEA1 in A1, B2, D1 cells. **D.** Karyotypes of A1, B2, and D1 cells. **E.** Embryonic bodies derived from A1, B2, and D1 cells. **F.** Immunofluorescence assay of markers of the three germ layers, including AFP, beta TUBLIN, and DESMIN, in EB differentiated cells. Scale bar, 50 μm for A, C. and F; 100μm for E.

Figure S2. Morphology of DOX-iPSCs grown in LF2i and 2i media at passage 5. Scale bar, 100 μm.

Figure S3. Growth of DOX-iPSCs in Dox-free 2i medium. **A.** Morphology and AP staining of DOX-iPSCs cultured in 2i medium with 0-4μg/mL doxycycline. **B.** Quantitative RT-PCR analysis of transgenes (*EX*) and endogenous pluripotent genes (*EN*) from DOX-iPSCs cultured in 2i medium with 0-4 μg/mL doxycycline. Data indicate mean ± SD. *P <0.05, **P <0.01, n=3. **C.** Diagram of DNA fragments located in 5'end Oct4 gene and 3'end c-Myc gene for qRT-PCR analysis of transgenes.

Figure S4. Morphology of DOX-iPSCs cultured in Dox-free 2i-plus medium with the different concentration of PD0325901 for 5 days. Scale bar, 100 um.

Figure S5. Diagram of the three reporter vectors with OCT4 enhancers that were used for the activation analysis.

Fig. S6. Morphology and AP staining of iPF4-2 grown in the reported medium. Scale bar, 100 um.

Fig. S1

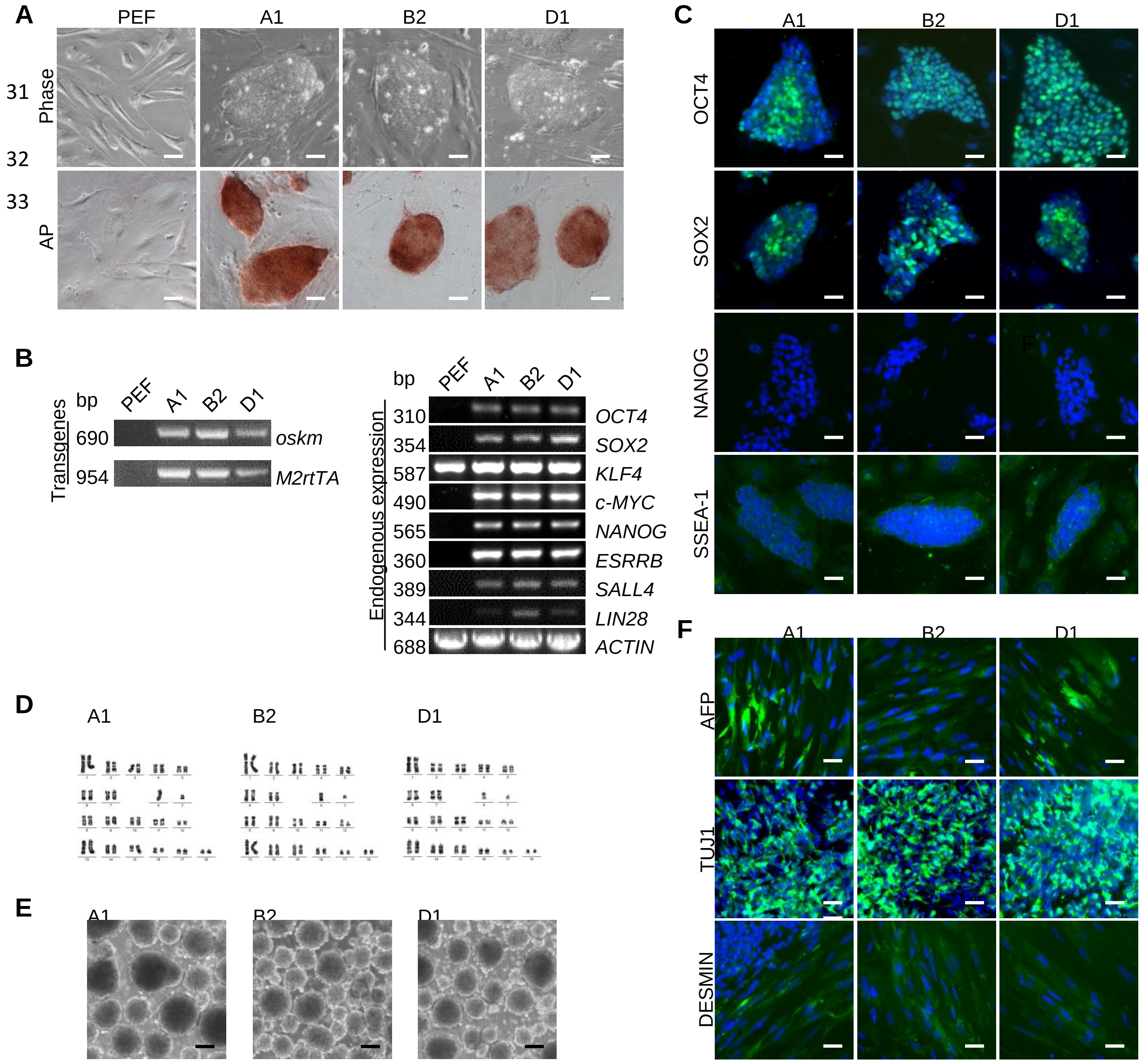
A
PEF
A1
B2
D1
Phase
AP
B
Transgenes
bp
PEF
A1
B2
D1
690
oskm
954
M2rtTA
Endogenous expression
bp
PEF
A1
B2
D1
310
OCT4
354
SOX2
587
KLF4
490
c-MYC
565
NANOG
360
ESRRB
389
SALL4
344
LIN28
688
ACTIN
C
A1
B2
D1
OCT4
SOX2
NANOG
SSEA-1
D
A1
B2
D1
E
A1
B2
D1
F
A1
B2
D1
AFP
TUJ1
DESMIN

Fig. S2

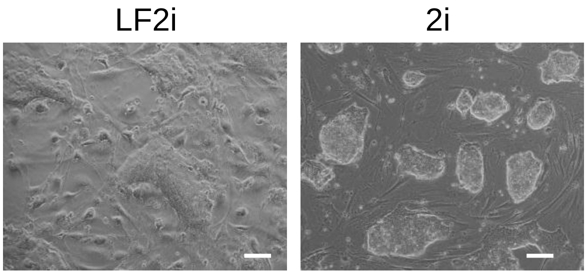

Fig. S3

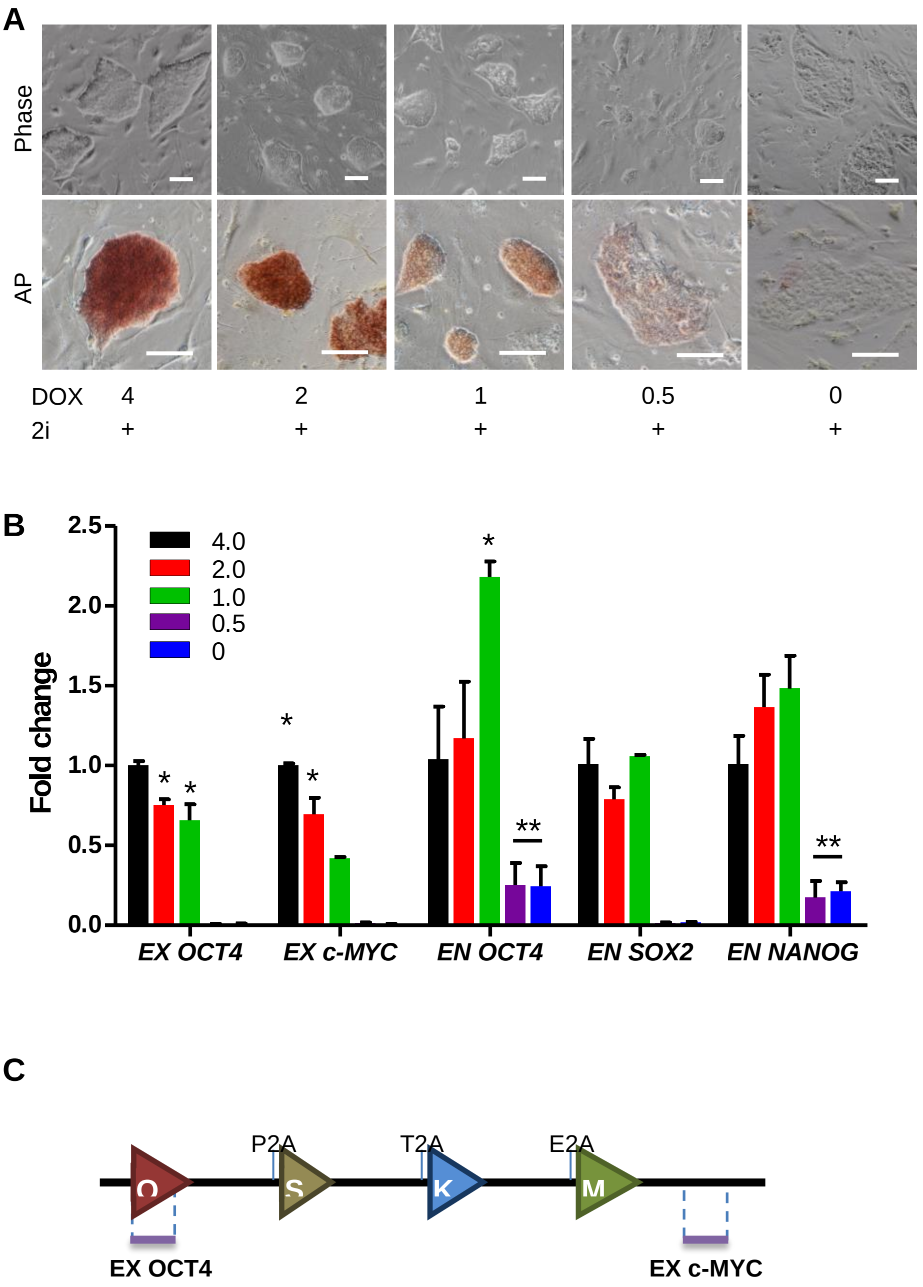
A
Phase
AP
DOX 4 2 1 0.5 0
2i + + + + +
B
Fold change
2.5
2.0
1.5
1.0
0.5
0.0
4.0
2.0
1.0
0.5
0
*
**
EX OCT4
EX c-MYC
EN OCT4
EN SOX2
EN NANOG
C
P2A
T2A
E2A
O
S
K
M
EX OCT4
EX c-MYC

Fig. S4

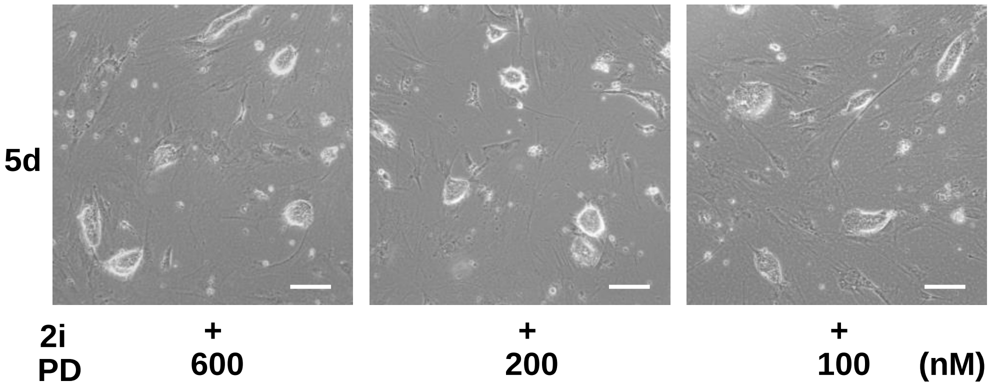

Fig. S5

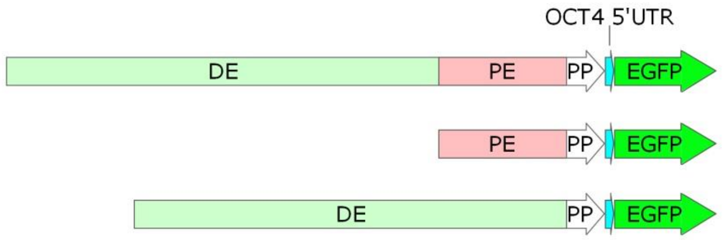

Fig. S6

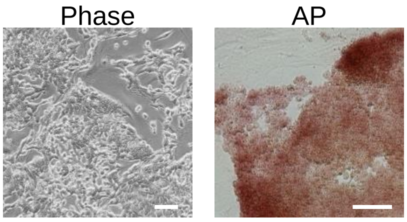

Supplementary Tables

Table S1 Media used in this study

Ingredients	Media											
	LF2i	LF	F2i	L2i	2i	2i+	3i	LFB2i	Xiao’s lab	MXV	LIF medium	bFGF medium
DMEM	√	√	√	√	√	-	-	√	-	-	-	-
DMEM/F-12	-	-	-	-	-	√	√	-	√	√	-	-
KO-DMEM	-	-	-	-	-	-	-	-	-	√	√	-
Neurobasal	-	-	-	-	-	-	-	-	-	√	-	-
mTeSR1	-	-	-	-	-	-	-	-	-	-	-	√
N2/B27	-	-	-	-	-	-	-	-	-	√	-	-
BSA	-	-	-	-	-	-	-	-	-	√	-	-
FBS	√	√	√	√	√	-	-	√	-	-	√	-
PL	-	-	-	-	-	√	√	-	-	-	-	-
KSR	-	-	-	-	-	-	-	-	√	√	-	-
Vitamin C	-	-	-	-	-	-	-	-	-	√	-	-
Forskolin	-	-	-	-	-	-	-	-	-	-	√	-
LIF	√	√	-	√	-	-	-	√	-	√	√	-
bFGF	√	√	√	-	-	-	-	√	-	√	-	√
BMP4	-	-	-	-	-	√	√	√	-	-	-	-
SCF	-	-	-	-	-	√	√	-	-	-	-	-
IL-6	-	-	-	-	-	√	√	-	-	-	-	-
EGF	-	-	-	-	-	-	-	-	-	-	-	-
CHIR99021	√	-	√	√	√	√	√	√	-	-	-	-
SB431542	√	-	√	√	√	√	√	√	-	-	-	-
PD0325901	-	-	-	-	-	-	√	-	-	-	-	-
Dox	√	√	√	√	√	√	-	-	√	-	-	-

Table S2 Vectors used in this study

Name	Function	Genes	Promoter
pCL-ECO	Retro-virus packaging	Virus elements	CMV
pCMV-VSV-G	Virus packaging	VSV-G	CMV
pMXs-EGFP	Retro-virus / Expression	EGFP	Virus
pMXs-hOCT4	Retro-virus / Expression	Human OCT4	Virus
pMXs-hSOX2	Retro-virus / Expression	Human SOX2	Virus
pMXs-hKLF4	Retro-virus / Expression	Human KLF4	Virus
pMXs-hC-MYC	Retro-virus / Expression	Human C-MYC	Virus
psPAX2	Lenti-virus packaging	Virus elements	chicken β-actin
pMD2.G	Virus packaging	VSV-G	CMV
TetO-FUW-OSKM	Tet-On element	m-OSKM	Tet-On
FUW-M2rtTA	Tet-On element	rtTA-Advanced	hUbC
pSIN-EF2-EGFP	Lenti-virus / Expression	EGFP / PuroR	EF-1a
PL-SIN-EOS-S(4+)-EGFP	Lenti-virus / Expression	EGFP / PuroR	Sox2 enhancer (x4)
pEGFP-1-OCT4-pro	Reporter	EGFP	OCT4
pEGFP-1-DEPP	Reporter	EGFP	OCT4 distal
pEGFP-1-PEPP	Reporter	EGFP	OCT4 proximal

51 Table S3 Primers used in this study

Name	Sequence	Gene	Size	Reference
p-ACTB-Q-161-F	GTGACAGCAGTCGGTTGGAT	beta-ACTIN	161	XM_003124280
p-ACTB-Q-161-R	TTTTGGGAAGGCAGGGACTT			
p-ACTB-P-688-F	GTGCGGGACATCAAGGAGAA	beta-ACTIN	688	XM_003124280
p-ACTB-P-688-R	GTCACCTTCACCGTTCCAGT			
p-POU5F1-PQ-310/179-F	**T**GAGGC**T**TTGCAGCTCAG**TT**	OCT4	310/179	NM_001113060
p-POU5F1-Q-179-R	TCTCCAG**GT**T**G**C**CT**CTCACT		179	
p-POU5F1-P-310-R	A**C**TGCTTGATC**G**TTTGCCCT		310	
p-SOX2-P-354-F	TAAGTACAC**A**CT**G**CCCGGAG	SOX2	354	NM_001123197
p-SOX2-P-354-R	CATGGA**A**CC**G**AGCG**T**CAT**G**C		354	
p-SOX2-Q-219-F	AG**C**AGACT**T**CACATG**T**CCCAG	SOX2	219	NM_001123197
p-SOX2-Q-219-R	TG**A**GA**G**AG**A**GGCAGTGT**A**CCG		219	
p-KLF4-P-587-F	TAGCAAAGG**C**AGCCCAGA**T**G	KLF4	587	NM_001031782
p-KLF4-P-587-R	GGGC**A**AATTTCCACCCACAG			
p-KLF4-Q-86-F	GAAGGGAGAAGACACTGCGT	KLF4	86	NM_001031782
p-KLF4-Q-86-R	CGGGGGAAGTC**TTTGCTTCA**			
p-C-MYC-P-490-F	AGGACTGTATGTGGAGCGG**T**TTC	c-MYC	490	NM_001005154
p-C-MYC-P-490-R	AG**T**GGGCTGTGCGGAGGTTT			
p-C-MYC-Q-188-F	AGAG**A**TGCCA**T**GTGTCCAC**G**	c-MYC	188	NM_001005154
p-C-MYC-Q-188-R	**A**TTGTGTGTCCGCCTCTTGT			
p-NANOG-Q-101-F	AGGGCTCAGCCAGTACAGAA	NANOG	101	NM_001129971
p-NANOG-P-565-F	TTGCCCCGAAGCATCCATT		565	NM_001129971
p-NANOG-PQ-565/101-R	CCAGCTCTGATTACCCCACA		565/101	
p-LIN28A-PQ-344/246-F	TGCCGGCATCTGTAAATGGT	LIN28	344/246	
p-LIN28A-Q-246-R	CTCTCGCTCCCAATGCAGAA		246	NM_001123133
p-LIN28A-P-344-R	GCAGTTTGCATTCCTTGGCA		344	NM_001123133
p-SALL4A-Q-217-F	CCCCAACACATCAACTCGGA	SALL4	217	NM_001114673
p-SALL4A-Q-217-R	ACTCGGCACAGCATTTCTCA			
p-SALL4A-P-389-F	TGAGATGGAAGGTCGAAGCA	SALL4	389	NM_001114673
p-SALL4A-P-389-R	TTCCGTCCGTCCCTAACAGA			
p-ESRRB-Q-149-F	ATGCCTCAAAGTGGGGATGC	ESRRB	149	XM_001928051
p-ESRRB-Q-149-R	TTTTAGTCAATGGCTTCTTCGCA			
p-ESRRB-P-360-F	TGAGATCACCAAACGGAGGC	ESRRB	360	XM_001928051
p-ESRRB-P-360-R	GAGAAGCCTGGGATGTGCTT			
p-STELLA-Q-114-F	GAAAGGGGTGAGGACGTTGT	STELLA	114	XM_021093127
p-STELLA-Q-114-R	CGAAATCGCTGCTCTCCTGA			
p-REX1-Q-147-F	CGCGTTAGCATGGGAAATGT	REX1	147	XM_005672653
p-REX1-Q-147-R	AAGGGCCTCTTGTGTCCATTC			
p-REX1-P-462-F	GGCATCTTTGACAGCCAACTT	REX1	462	XM_005672653
p-REX1-P-462-R	TCTTCCACGAGGGGTTCAGA			
p-THY1-P-358-F	TCCCACCCTTGGTGAAAACT	THY1	358	XM_005667396
p-THY1-P-358-R	GTTCGAGAGCGGTAGGAGTG			
p -OTX2-Q-149-F	CACTGTTTGCTAAGACCCGATACC	OTX2	149	XM_021102521
p-OTX2-Q-149-R	GACCTCCATTCTGCTGTTGTTGC			
TetO-FUW-OSKM-F	**CACGCTGTTTTGACCTCCAT**	OSKM	690	
TetO-FUW-OSKM-R	TCTCATTGTTGTCGGCTTCCT			
FUW-M2rtTA-F	ACTCGGTTTGTCGTCTGT	M2rtTA	954	
FUW-M2rtTA-R	GCTACTTGATGCTCCTGTT			

Name	Sequence	Gene	Size	Reference
p-DE-F	TGCCAAGGAGAGGGAGCTAT	Distal enhancer	3152	
p-DE-R	CACATTGGGCCCCCACTGGGACCTA			
p-PE-F	GCCCTGTCTGAGGCTCAGT	Proximal enhancer	897	
p-PP-F	GCTGTCGGGGAGCCAGGT	Proximal promoter	268	
p-PP-R	GGGGAAGGAAGGCGCCCCAA	M2rtTA	954	
Oskm/oct4-F	CTGGCTTC**A**GACTTCGCCT	OCT4	142	
Oskm/oct4-R	CCTCTG**A**GCC**T**GGTCC**GATT**			
Oskm/MYC-F	GCT**T**CG**A**AACTCT**G**G**TG**CAT	c-MYC	185	
Oskm/MYC-R	**ATGAAAGCCATACGGGAAGCA**			

Note: p, porcine specific primer; P, primers are used for RT-PCR; Q, primers are used for qRT-PCR. Those **bold and underlined** letters are species specific bases for either exogenous mouse genes or endogenous porcine genes.
